# Case Report: Enhancing lead extraction techniques: a novel approach using a loop formed by an ablation catheter and a gooseneck snare

**DOI:** 10.3389/fcvm.2024.1361370

**Published:** 2024-02-27

**Authors:** Jun Li, Lei Wang, Xiaofang Li, Na Li, Xin Shao, Anxin Zhang, Yan Shu, Haixiong Wang

**Affiliations:** ^1^ Department of Cardiology, Shanxi Cardiovascular Hospital, Taiyuan, Shanxi, China; ^2^ Department of Digestive Oncology, Shanxi Bethune Hospital, Tongji Shanxi Hospital, Shanxi Academy of Medical Sciences, Third Hospital of Shanxi Medical University, Taiyuan, Shanxi, China

**Keywords:** pacemaker, lead extraction, very old lead, infection, catheter, loop

## Abstract

The difficulty and complexity of lead extraction procedures increase with the age of the lead to be extracted. The extraction of old (>20 years) leads is more time-consuming and requires advanced tools and a complex technique. In this case, we retrieved a very old (>30 years) lead using a loop formed by a catheter and a gooseneck snare. The catheter was rotated to remove the lead-bound sites. The lead was successfully retrieved using a Needle's Eye Snare.

## 1 Introduction

Although several techniques have been developed, successful pacemaker lead extraction is still not achieved in approximately 2.6% of cases (1); most of these cases ultimately require surgical intervention. Therefore, it is crucial to develop more effective extraction techniques. We present a novel technique that employs a loop formed by a catheter and snare to facilitate lead removal.

## 2 Case description

**Figure 1:**
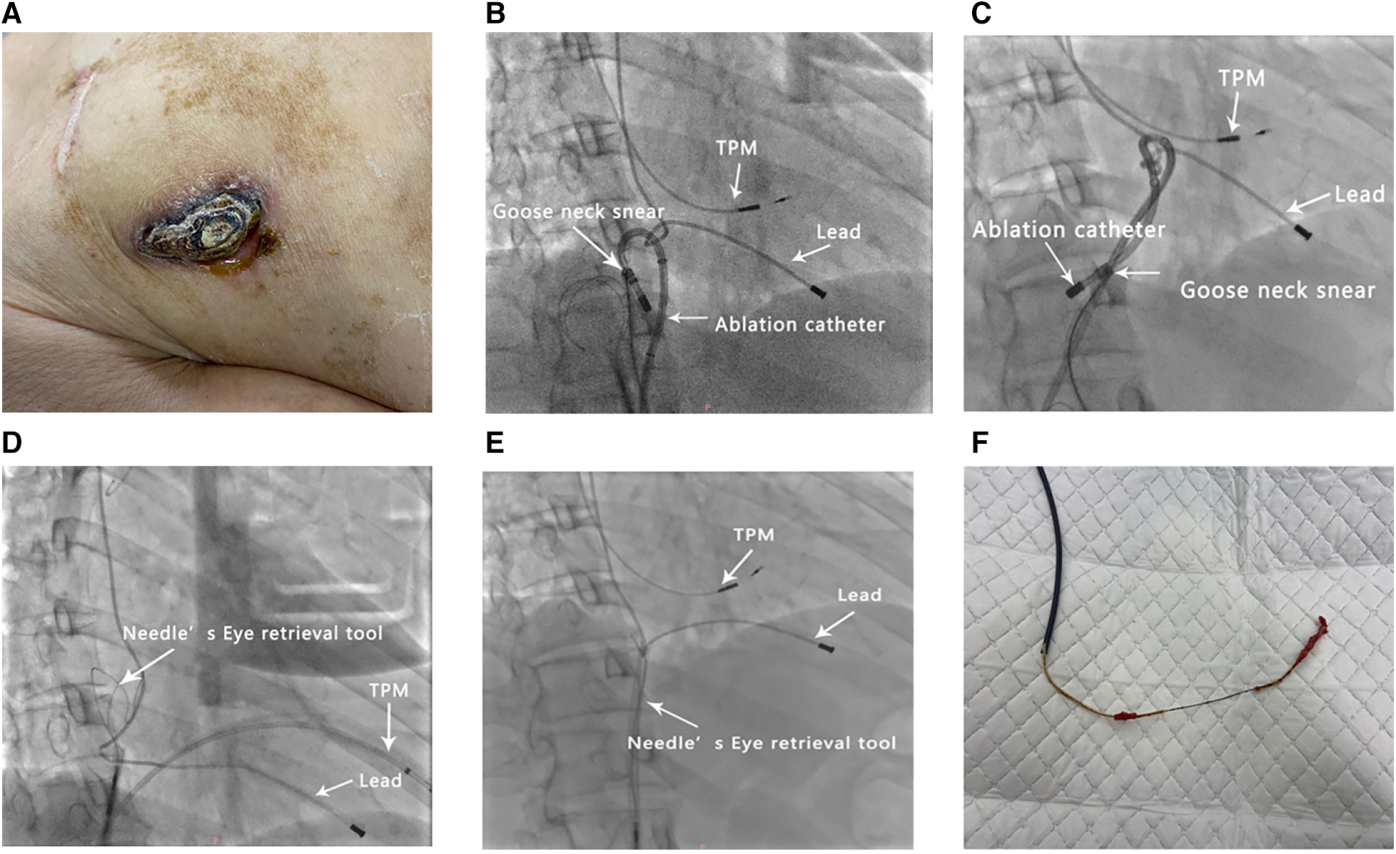
Illustration of the procedure. (**A**) A pacemaker that has eroded through the skin. (**B**–**F**) Serial fluoroscopic images (right anterior oblique) showing (**B**) the retrieval of the lead with a loop formed by a catheter and gooseneck snare, (**C**) rotation of the catheter, (**D**) removal of the lead-bound sites and preservation of the slackness of the lead, (**E**) retrieval of the lead using a Needle's Eye Snare, and (**F**) calcified fibrotic adhesions on the extracted lead. TPM, temporary pacemaker.

A 53-year-old female with a history of symptomatic complete atrioventricular block formerly treated with permanent pacemaker implantation was admitted for the management of an eroded and infected pacemaker pocket. The initial single-chamber pacemaker implantation was performed in the right side of the chest in 1992. The functioning of the pacemaker was reviewed in 2017; new atrial and ventricular leads and a new dual-chamber pacemaker generator were implanted. The initial ventricular lead was defective but was abandoned and left in position. Notably, the pacemaker eroded through the skin, with evidence of purulent discharge in August 2022. She was afebrile and on antibiotics; no fever was documented before presenting at the hospital. The right anterior chest shows thinning and necrosis of the tissue at the inferior margin of the pacemaker. There was hemorrhagic tissue with mild erythema and discharge but no noticeable odor (Figure 1A). The laboratory profile revealed a leukocyte count of 11.58 per mm^3^ (3.50–9.50 per mm^3^), an NT-proBNP concentration of 3,451 ng/L (0–125 ng/L), and a CRP concentration of 63.7 mg/L (0.0–8.0 mg/L), and multiple blood cultures were negative but the wound secretion culture revealed *Staphylococcus aureus* after 72 h. Echocardiography was used to identify the right ventricular leads, and no significant vegetation was observed on the valve or lead. The procedure was performed under deep sedation with midazolam and propofol in an electrophysiology laboratory with continuous arterial blood pressure and oxygen saturation monitoring. In cases of pacemaker dependence, temporary pacing was performed via the left femoral vein.

After removing the generator and exposing the lead, a stylet was placed in the atrial lead, the screw was retracted, and the lead was removed with gentle traction. A stylet was placed into the screw-in ventricular lead; the screw was retracted, and the lead was extracted into the subclavian vein with sustained traction. Densely calcified adhesions around the remaining passive-fixation ventricular lead were observed by transesophageal echocardiography at a local hospital, which cannot be dissolved with laser techniques. After discussion, it was decided that a mechanical approach should be performed in this patient. Subsequently, the lead was sized with a locking stylet, and a 9-french EvolutionRLTM (Cook Vascular Inc., USA) was used to clear the entry point of the lead into the subclavian vein beneath the clavicle; however, this was not achieved. Next, a 16-french sheath workstation was placed via the right femoral vein, and a Needle's Eye Snare (Cook Medical) was used to retrieve the lead; nevertheless, subsequent attempts to retrieve the lead were unsuccessful. During the procedure, an intermittent pacemaker malfunction occurred; therefore, a new 5076-58 lead (Medtronic, USA) was screwed into the myocardium via the left subclavian vein for stable cardiac stimulation. After lead implantation, fibrosis and binding sites can develop between the leads, vessels, and heart (2).

The inflammatory process continued after implantation, leading to extensive fibrosis, calcification, and ossification of interface segments between the vein and lead (3). Several attempts were made to retrieve the lead using the loop formed by a wire and snare, but these failed. Given the good maneuverability of the catheter [Biosense Webster ThermoCool SmartTouch(ST)], we successfully retrieved the lead using a loop made up of a catheter and a 5-F loop snare catheter (Shanghai Shape Memory Alloy, Shanghai, China) (Figure 1B). Rotation of the catheter enabled the removal of the lead-bound sites (Figures 1C, D). Finally, the Needle's Eye Snare was used to retrieve and extract the lead (Figures 1E, F). The operation time was 150 min and the fluoroscopy time was 46 min.

Ventricular ectopy or coagulum formation was not observed, and the procedure was well-tolerated. Subsequently, a leadless pacemaker (Micra^TM^ AV-TPS, Medtronic Inc., Fridley, MN, USA) was placed in the right ventricle 1 week later to reduce the risk of pocket- and lead-related complications after the completion of antibiotic therapy, and multiple blood cultures were negative. The patient remained afebrile with no further symptoms or signs of infection and all device parameters remained stable during follow-up.

## 3 Discussion

**Figure 2:**
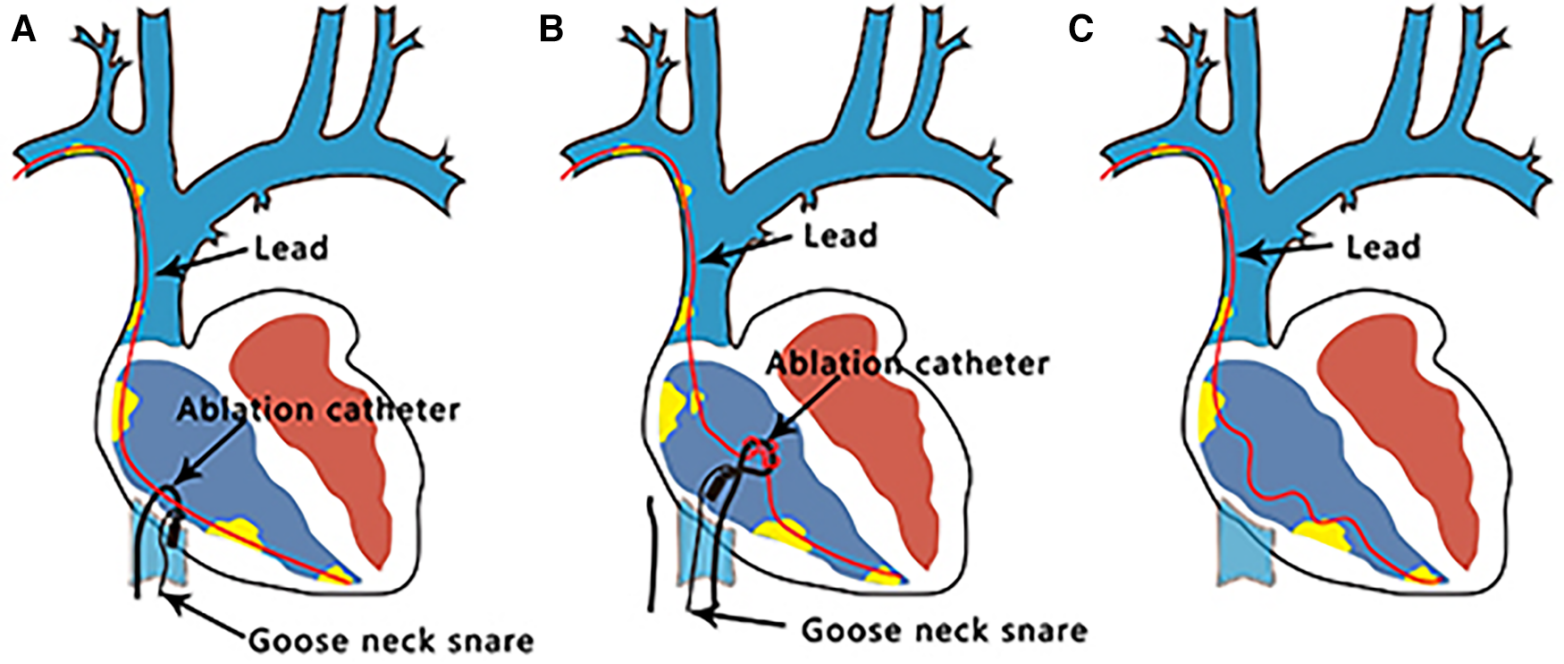
Lead extraction process. (**A**) A loop is formed using a catheter and gooseneck snare. (**B**) Catheter rotation induces the removal of the adhesions around the leads. (**C**) Sufficient local slack was achieved for complete lead removal using a Needle's Eye Snare.

Transvenous lead extraction (TLE) is a vital procedure for patients with cardiac implantable electronic devices (4). Using advanced instruments, the extraction of very old leads can be effectively and safely accomplished (5); even same-day discharge can be achieved for uncomplicated cases (6). Radiofrequency (RF) energy delivered with a steerable ablation catheter to facilitate lead removal has been reported in several cases (7, 8). However, using an ablation catheter to form a loop to remove lead-bound sites has not yet been reported. Given the need for successful lead extraction in the presence of an infection and the relative failure rates of percutaneous extraction with current techniques, it is crucial to improve the technical approach and develop new technologies to facilitate extraction. Our case provides a potential strategy (Figure 2); however, this technique should be used cautiously, and its inherent risks (tamponade, valve damage, etc.) must be understood.

## Data Availability

The datasets presented in this study can be found in online repositories. The names of the repository/repositories and accession number(s) can be found below: not applicable.
